# The practical year: a qualitative study on stressors, resources and proposed improvements among medical students

**DOI:** 10.1186/s12909-025-07788-2

**Published:** 2025-08-23

**Authors:** Lisa Guthardt, Syna Franck, Thomas Muth, Adrian Loerbroks

**Affiliations:** Institute of Occupational, Social and Environmental Medicine, Centre for Health and Society, Medical Faculty, Heinrich Heine University Düsseldorf, Moorenstr. 5, 40225 Düsseldorf, Germany

**Keywords:** Germany, Medical students, Medical education, Teaching, Psychological well-being, Qualitative research, Psychological stress

## Abstract

**Background:**

The last academic year in medical faculties in Germany is the so-called practical year (PY), which is a combination of studying and on-the-job clinical training. Based on research documenting considerable stress among both medical students and resident physicians it can be assumed that PY students likewise experience high levels of stress. However, evidence remains sparse for medical students in the practical year in Germany and elsewhere.

**Methods:**

PY students at a German medical school were recruited via purposeful and snowball sampling and semi-structured qualitative interviews were conducted by telephone. Data collection was carried out until data saturation was reached. The interviews were voice-recorded, transcribed verbatim and content-analyzed using MAXQDA software (2020).

**Results:**

Eighteen female and seven male participants (*n* = 25; mean age: 26.6 years; standard deviation (SD = 3.7)) were interviewed (mean duration = 36 min with SD = 7.6). Mentioned stressors included a lack of skills and knowledge, poor supervision, routine tasks (i.e., lack of learning experiences due to repetitive work), lack of appreciation (mainly on the part of superiors) and a heavy workload. Resources included working with patients and their appreciation, a positive learning and working atmosphere on the wards, learning and knowledge acquisition, one-on-one supervision by a medical colleague, clinical-practical training as well as a positive work-life balance with sufficient leisure time. Among other things, the PY students suggested a better pay and a change in absence regulations as well as the opportunity to take care of their own patients and to have structured PY teaching.

**Conclusion:**

PY students perceived multiple stressors, but also resources and suggested potential improvements. There is a subjective need for improvements of the working and training conditions in the PY. However, further studies are required to quantify and prioritize those needs and to explore the feasibility of the suggested interventions.

**Supplementary Information:**

The online version contains supplementary material available at 10.1186/s12909-025-07788-2.

## Background

The distress of medical students and clinically active physicians has received research attention for many years. Medical students experience numerous stressors such as a high workload, fear of failure and feeling unprepared for clinical work as well as the feeling of social isolation during examination phases and organizational challenges [1–3]. Resident physicians also experience various stressors such as limited autonomy, stressful relations with supervisors and added responsibility for patient care [4] as well as concerns related to mistakes and poor patient outcomes [5].

The so-called practical year (PY) is part of medical education in Germany and represents the clinical-practical training period in the final year of medical studies. It is a training phase at the clinic that lasts a total of 48 weeks, is divided into three tertials (i.e., internal medicine, surgery, one elective) of 16 weeks each. The PY can be seen as a transition period between being a student and being a resident physician (for more details on the practical year in Germany see Appendix A). Similar extended clinical periods at the conclusion of medical studies are also incorporated in medical curricula elsewhere, e.g., in the USA [6] and various European countries (e.g., Belgium, the Netherlands and Ireland [7]). Stressors, psychosocial resources and suggestions for improvement in terms of preventive measures have already been well-investigated among medical students [1, 2, 8, 9] and resident physicians [5, 10–12]. In contrast, evidence from PY students is less extensive: Surveys [13, 14] and qualitative studies with PY students in Germany [15–17] and abroad [3] have identified several stressors such as a lack of feedback from supervisors, work overload with routine activities, worries about the future, poor work-life balance and financial worries. The psychosocial resources included, amongst others, support from family and doctors [3, 15, 17], and potential improvements have been explored (e.g., regarding further training) [15, 16, 18]. Notably, most of the prior evidence stemmed from the pre-COVID-19 pandemic era. The COVID-19 pandemic had a great impact on both work in the healthcare sector [19–22] and medical studies [23–26]. Students may feel barely prepared for the final year, as most of their clinical training in medical school before the PY took place online (e.g., minimal patient contact, no opportunities to acquire practical skills). Therefore, it remains elusive to what extent studies before the pandemic capture the distress experience of PY students in the post-COVID-19 era. Even though there is considerable empirical evidence on medical students’ distress and individual aspects concerning the final year, we wanted to provide an in-depth picture of the learning situation of medical students in their PY. Our objective was therefore to not only focus on perceived stressors and resources but also include improvement suggestions by the students themselves. We combined these three topics in a qualitative study to gain in-depths insights from the persons affected focusing on the transition phase from a more theoretical learning environment to practical training in the clinic.

## Methods

We adhered to the consolidated criteria for reporting qualitative research (COREQ) [27].

### Research team

The research team consisted of a doctoral student who was herself a medical student at the time of study planning, data collection and analysis and writing of the paper (SF), a researcher experienced in qualitative research (LG; [28, 29]) and two senior researchers (TM and AL) who have previously researched stress research among medical students using qualitative methods [1, 2, 8]. SF conducted the interviews under the supervision of AL and TM.

### Study design and recruitment

The study applied a qualitative descriptive design and was carried out using content-structuring qualitative analysis according to Kuckartz [30]. With regard to the inclusion criteria, study participants had to enrolled as medical students at the Heinrich Heine University (HHU) Düsseldorf, Germany, and had to be at the beginning of the second tertial of their PY (11th semester) at the time of data collection. We chose this time point, because we assumed that participants already had some experience (compared to the first tertial), but had not become fully used to the PY yet (compared to the third tertial). Sufficient knowledge of German was also mandatory in order to be able to participate. The students were located in Germany or abroad at the time of data collection and were recruited from the 2022 fall cohort, which included 180 PY students. Participants were recruited from a WhatsApp group called “PY – fall 2022” with 184 members. Overall, we mainly followed a purposeful sampling strategy but also combined different approaches (also convenience and snowball sampling), which is common in qualitative research [31]. There were two recruitment phases: At first, interviews were conducted with all participants who were interested after an initial call for participation (chronologically depending on their response and availability). Since more female students expressed an interest to participate, there was a second call in the same group chat specifically calling on male students. All male students who contacted SF were then also included. During both phases, participants were also encouraged to contact other students who might be interested in participating. Participants received financial compensation of 30 euros. Ethical approval was obtained from the Ethics Committee at the Medical Faculty of the Heinrich Heine University prior to data collection (# 2019 − 510_5) and PY students provided informed written consent prior to their participation.

### Data collection

SF conducted semi-structured telephone interviews based on a topic guide between mid-March and early April 2023. The topic guide was based on prior experiences from members of the research team as well as results from a previous qualitative study [15]. It was discussed several times within the study team until consensus was reached, was reviewed and further adapted during data collection. The guide included a general introductory question (“How have you experienced the PY so far?”), and questions on perceived stressors, resources, coping strategies and improvement suggestions (e.g., “What stresses you in your day-to-day work?” or “What would you like to change if you had the opportunity?”). Additionally, demographic information was inquired (e.g., gender, year of birth, semester), all on a voluntary basis (see Appendix B for the complete and final version). After four interviews, AL listened to the audio files of the recorded interviews and provided SF with feedback relating to her conduct of the interviews. The interviews were conducted until it was felt that data saturation was reached, which seemed to be the case after 18 interviews. Seven more interviews were conducted to verify this assessment. The interviews were transcribed. Transcripts were not sent back to the participants for comments and/or corrections.

### Data analysis

The interviews were content-analyzed [30] using MAXQDA (version 2020, VERBI GmbH, Berlin, Germany). Two members of the research team (LG and SF) independently coded the first 12 interviews. The code systems were discussed until consensus was reached and merged. The resulting coding system was used by SF to code the remaining interviews and was expanded during that process. The coding system and coded segments were repeatedly checked by LG and AL during the analysis. AL reviewed the entire coding system again after all interviews had been coded. It was then minimally revised, and all interviews were coded again by SF. This coding round was considered final. A combined deductive-inductive approach was chosen for the coding: the three main categories (stressors and resources as well as suggested solutions from the perspective of the PY students) were formed based on the research question and the topic guideline. First subcategories were also created deductively based on results from a previous qualitative study on stress in final year medical students [15]. Further main categories and subcategories then inductively emerged from the material in the process of analyzing.

## Results

### Sample description

Eighteen study participants reported to be female and seven to be male (*n* = 25; 23–38 years; Ø 26.6 years; standard deviation [SD] = 3.7, see Table 1). The interviews lasted an average of 36 min (SD = 7.6). On average, the students worked 37.1 h/week (SD = 4.8). As much as 44% of participants began their elective traineeship, while 28% of participants were in surgery and 28% were in internal medicine at the time of data collection. More than half of the respondents were in an academic teaching hospital (52%), around a third (36%) in a university hospital and just under 12% reported to be located abroad.

**Table 1:** Sample description (*n* = 25)

Characteristics	
Age (years); mean (SD^a^)	26.6 (3.7)
Gender, *n*	
Female	18
Male	7
Interview duration (minutes), mean (SD)	36 (7.6)
Working hours (incl. overtime; hours/week), mean (SD)	37.1 (4.8)
Current rotation, *n*	
Elective tertial^b^	11
Surgery	7
Internal medicine	7
Current location of the rotation, *n*	
Academic teaching hospital	13
University hospital	9
Abroad^c^	3

^a^ Standard deviation

^b^ Anesthesiology, radiology, radiotherapy, urology, neurology, ear, nose and throat medicine, dermatology

^c^ Switzerland, South Africa

### Results from the qualitative interviews

Findings are presented arranged by the three main categories (i.e., stressors, resources and suggested improvements). Selected quotes are presented in the text. Additional quotes can be found in the tables and the appendix. The quotes were translated by LG, who holds a master’s degree in Literary Translation.

### Stressors

A summary of different stress factors mentioned by the students sorted by main categories with a selection of subcategories and exemplary quotes can be found in Table 2.

**Table 2:** Stress factors (main categories with a selection of subcategories and exemplary quotes)

Categories	Subcategories	Exemplary Quotes
Learning and working environment	Lack of appreciation and collegial behavior (e.g., feeling like a lackey)	“Well, there were some wards or departments where they didn’t include you at all. You were just the resident physician idiot doing all the tedious work. And you also had to run some errands that were really unnecessary.” (Interview 21)
	Poor/negative interaction with each other (e.g., arguments, bad talking, harsh tone)	“[…] Senior physicians or the chief physician who didn’t ask what my name was during the whole tertial, even though I was assisting them in surgery almost every day. And for me, this was most stressful, that I was completely ignored and overlooked.” (Interview 25)
	Hindered learning (e.g., no room for questions or mistakes)	
Teaching	PY seminars/courses (e.g., structure, format, scheduling, topics, absences)	“There were PY seminars every Friday where the doctors would randomly talk about certain topics, provided that they showed up at all. The seminars aren’t really structured. For example, one surgeon tells you: ‘Alright, let’s talk about colon carcinoma.’ And the next day, there is another surgeon telling you: ‘Well, I have prepared colon carcinoma for today […] Oh well, what else do you want to do?’ Most seminars were like that, and I think that wasn’t very useful.” (Interview 1)
	Poor supervision (e.g., lack of training, missing medical contact person or supervisors)	
Work-related aspects	Emotional stress/death (e.g., patients dying, lack of contact persons to address emotional stress)	
	Work content and processes (e.g., non-medical tasks, routine tasks, no perceived increase in competence, interruptions, working with machines/programs, no own workplace)	
	Skill level and autonomous working (e.g., fear of making mistakes, practical uncertainties, no or little independent work, sitting around, no own patients, too much or too little responsibility, workload too high or too little)	“It happens quite often that you are basically alone on the ward because all doctors are in the operating room or busy with other examinations or whatever. And then you’re the person responsible in case of an emergency. And this responsibility really scares you. I personally do not feel ready to properly deal with that.” (Interview 16) “Last week, it was half past four already and a patient hadn’t woken up from anesthesia yet, it just took a lot of time. And then, the resident physician I was working with said: 'Alright, I’ll leave the PY student with you, I’m going home now.'” (Interview 23)
Work organization	Bureaucracy and guidelines (e.g., no employment contract with a clear number of working hours per week)	“So, you don’t really have an employment contract or internship contract, but everything is quite blurry in the organization.” (Interview 11)
	Lack of work clothes	
	Ward capacities and rotations (e.g., too many students per ward, new supervisors and environment due to rotations)	“We were seven people and there were only about three or four surgeries per week for all of us.” (Interview 4)
	Staff shortage	
Social interactions	Inter- and intraprofessional interaction (groups of people: nursing staff, doctors, patients/relatives, other PY students)	“During ward rounds, there were four senior physicians and three assistants rushing in there and they pulled the plasters off the patients’ wounds somehow, they didn’t even really look at them. They just quickly put on a new plaster and the patient didn’t even have a chance to tell them how he was feeling. And then, the doctors rushed out again and I often stayed in the room because I noticed that the patient still had some questions.” (Interview 21)
	Team integration, hierarchies and expectations (e.g., poor integration in the team, strong perceived hierarchies, expectations from doctors and nurses too high with regard to what PY students can do).	“It’s a very hierarchical structure. So, the chief physician makes it very clear that he is the chief physician, and the senior physicians make it very clear that they are senior physicians. Things are carried out via the assistants, even though they are not responsible. But you can really tell that the stress is passed on from the top down in a way. And those who are least responsible for it actually take the brunt of it.” (Interview 5)
Time	Shift work/working hours (e.g., lack of break times, working on weekends/public holidays, early start of shift, no daylight, overtime/late end of shift)	
	Absence regulations and lack of study days (e.g., no differentiation between holidays and sick days)	“However, I think it’s a bit stupid that sick days are also included. Especially this semester, because the rule of isolation due to covid has been dropped, so it no longer counts as an excuse but is also included in the days of absence. In general, I think it’s annoying that every employee is naturally allowed to be sick, and we are not, or it is imposed on us later on, when we want to prepare for the third state examination.” (Interview 14)
Finances	Low expense allowance	“I think the payment is extremely awful. Sometimes, you really have gratification crises. You’re thinking about the fact that you spend nine or ten hours a day here, you also stay longer to help others. And at the end of the month, you don’t really see that. Instead, you have to try to get enough money. That was really stressful for me. Because you also compare your situation with people who have other jobs, and they don’t have to work that much and get more money for it.” (Interview 3)
	Covering living costs (e.g., need to get a part-time job in addition to studying)	
Personal matters	Thoughts and plans about the future (e.g., PY clinic as potential first employer, fears/worries about the future regarding the upcoming choice of specialist and work as a resident physician	“I also have great respect for the residents who are doing this on a regular basis. And yeah, I’m also a bit scared of that [own residency].” (Interview 17)
	Negative work-life balance (e.g., through commuting, doctorate)	“Fun things, like meeting your friends or doing sports, really miss out in a way.” (Interview 22)
	Character traits	“I don’t really push myself into the foreground. And when I always have to run after people so that you’re able to do something, that’s just a bit difficult.” (Interview 7)

#### Learning and working environment

Students perceived a lack of appreciation regarding their performed work by doctors, nurses and/or patients and a general lack of collegial behavior among colleagues or fellow students (e.g., no mutual help because every student seems to be fighting for themselves). That way, they sometimes seemed to feel like inferior workers:

“You’re feeling like some kind of lackey who is doing someone’s dirty work. And you don’t really feel appreciated that way.” (Interview 10).

They also perceived social tensions in the ward teams, e.g., arguments or being talked to in a harsh tone or a rough treatment by doctors and nursing staff. Additionally, students talked about factors that hindered their learning, as there often seemed to be no room for questions or mistakes:

“Nursing staff who would scream at you for no apparent reason, just because you were too slow. Or they asked you why you were so stupid and missed that vene in blood sampling.” (Interview 1).

Overall, this seemed to create a negative learning and working environment for them.

#### Teaching

Throughout the entire PY period, teaching events and time for self-study are supposed to be scheduled [32]. A lack of structure, an arbitrary choice of topics and inadequate scheduling of PY teaching units were perceived as stressful (e.g., frequent repetition of topics due to bad scheduling, short-term cancelling of lessons), as was the frequently used format of frontal teaching.

Participants sometimes felt poorly supervised as teaching doctors were felt to show little to no initiative regarding teaching content, and to not support the students or treat them with complete indifference. PY students reported a lack of fixed contact persons and inexperienced, overworked or stressed contact persons (usually resident physicians):

“What really bugs me at the surgical ward is the fact that you don’t really have any contact person at all. […] Nobody feels responsible for you. This means you’re just standing around somewhere, feeling lost and you hope that someone takes pity on you, takes you with them and guides you through the daily work routine.” (Interview 5).

These factors led to a perceived lack of training and preparation for their future jobs for them.

#### Work-related aspects

Participants perceived a variety of aspects related to work contents and practical work. Among other factors, they mentioned emotional stress related to experiences of death and dying. This seemed to be especially severe when they were confronted with death in the clinic for the first time and/or when they did not feel adequately prepared (e.g., when they have had no seminars on how to cope with death and dying):

“And then I had to reanimate the patient […]. And this adrenaline […] and the feeling afterwards because she died. […] That was the first time a patient died in my hands. And […] I was mentally overwhelmed in that moment.” (Interview 4).

Other aspects were related to tasks and work processes, e.g., the perceived need to take care of too many non-medical tasks such as running errands (e.g., bringing things to other wards or across the clinic area) or driving patients, many routine tasks per day such as blood sampling or difficulties related to technical devices and programs (e.g., no introduction so that students could not use them). Participants generally seemed to want more challenging tasks and more responsibility as they often felt underchallenged by routine tasks or non-medical tasks. However, if they were given new or too many tasks or (too) much responsibility, they quickly seemed to feel incompetent and overwhelmed as well. For the students, it was especially important to be adequately supervised instead of being left alone in challenging situations as this seemed to create an additional burden.

#### Work organization

For the students, a lack of uniform guidelines and standards in the PY could lead to stress, e.g., as there usually seemed to be no employment contract so that the number of working hours per week and the daily working time was not determined but more of a recommendation by the university and thus not binding for the clinics or wards.

Within a tertial (e.g., internal medicine) students usually rotate to different wards or departments (e.g., cardiology, nephrology). In this context, some students spoke of stressors related to the ward capacities, i.e., too many students per ward so that there was not enough work to do and they had to compete for tasks or too few so that they were missing exchange and mutual support, as well as too frequent or too few rotations. 4-week rotations were usually considered too short to develop a routine and to get to know their colleagues and superiors. Some PY students also talked about the lack of fixed rotation plans. They were assigned to new wards every morning and did not know in advance where and with whom they would be working that day.

PY students who worked on understaffed wards or departments seemed to be particularly stressed. In some cases, PY students felt that they were employed as full-time medical staff. They reported having to carry out routine tasks on several wards, sometimes requiring several hours of work and leaving no time for sufficient patient contact, teaching or interesting activities with the provided learning opportunities.

#### Social interactions

Inter- and intraprofessional interaction (e.g., between nurses and doctors or between doctors from different departments and hierarchical levels) was sometimes experienced as tense and uncooperative. Students often perceived a harsh tone and an emphasis of hierarchical structures. Furthermore, according to the participants, nurses and doctors were often unaware of the tasks that could be carried out by PY students and therefore had too high expectations.

Some students were also disappointed because the role of PY students seemed to be rather unknown in the general population and patients seemed to have little trust in the students’ abilities compared to those of the ward doctors. The lack of recognition of performance as a PY student seemed to be particularly evident in the interaction between female students and patients:

“I had a student apprentice with me on the ward and he talked with the doctor a lot. And I was the person who was being interrupted, or patients showed me pictures of their animals or children, even though I was doing an anamnesis interview. […] There are three people wearing gowns, two of them are male, one is female. And the female is the one they address with trivial issues and all the questions and important things are left to the men. And that really annoys me.” (Interview 13).

#### Time

Some students mentioned difficulties with working hours and shift work (e.g., being tired after night shifts, a lack of breaks or long shifts). They were also stressed by official absence regulations as they are not allowed to miss more than 30 days in the whole practical year (a maximum of 20 thereof in one tertial), regardless of whether these were sick or vacation days. This seemed to result in students feeling they were not allowed to be sick and sometimes neglecting their health (i.e., going to work even though they were not feeling well). Additionally, students said they tried to save 20 of those days for the third medical state examination (M3).

PY students also criticized the lack of study time they have in a week. On average, students said that they worked above the 20–25 working hours per week as prescribed by study and examination regulations. Students had the feeling that there was hardly any time to catch up on topics or adequately prepare ward rounds for the following day at home. Some hospitals did not seem to offer study days, i.e., days on which students can use their free time to study.

#### Finances

PY students reported on the low expense allowance, which was perceived as unfair compared to other professional groups in training phases (including nursing trainees) and resident physicians:

“I calculated it. My first tertial was my elective tertial, so I really enjoyed working there, but I did about 40 or 45 hours per week, and I got around 543 euros as an expense allowance, so it was an hourly wage of about 2 euros and 60 cents or even less.” (Interview 10).

In addition, there were considerable differences between hospitals and (federal) states according to the PY students.

#### Personal matters

Further individual stressors included thoughts and plans about the future (e.g., pressure to perform as the PY clinic might potentially be their first employer or fear of even more stress when being a resident physician), a negative work-life balance (e.g., due to commuting or the additional burden when doing a doctorate) and stress enhanced by certain character traits (e.g., more silent people have difficulties in standing out, feeling incompetent or like a burden to others).

### Psychosocial resources

A summary of different psychosocial resources mentioned by the students sorted by main categories with a selection of subcategories can be found in Table 3.

**Table 3:** Psychosocial resources (main categories with a selection of subcategories)

Categories	Subcategories
Learning and working environment	Pleasant atmosphere and recognition (e.g., fun at work, pleasant company of colleagues, friendliness)
	Learning is supported and encouraged (e.g., room for questions and mistakes)
Teaching	Good seminars/further training (e.g., students can express their own interests and make suggestions regarding the content of PY sessions, the seminars are well-structured and prepared)
	Adequate supervision in teaching (e.g., enough support, instructions/guidance, people who challenge and encourage the students, who are committed to and interested in students’ learning success as well as one-on-one teaching or mentoring)
Work-related aspects	Interesting work contents and good workflow (e.g., perceived knowledge gain, colleagues take over tasks that were not finished by the students, routine tasks to improve skills)
	Autonomy and responsibility (e.g., being able to work independently, doing practical work, having your own patients, feeling adequately prepared for the career start, being allowed to have responsibility but not having too much responsibility)
Work organization	Clear guidelines and expectations for PY students (e.g., communicated by supervisors in advance)
	Work equipment (e.g., work clothes, break room with fruits, own telephone)
	Medical contact person to address
	Division and structure of the tertial (e.g., adequate rotations, a structured training program, adequate ward capacities such as 1–2 students per department
Social interactions	Good inter- and intraprofessional interaction
	Nice team integration, good cooperation and flat hierarchies (e.g., adequate communication, mutual understanding)
Time	Shift work/working hours (e.g., knowledge gain in night shifts, compensatory days off, regulated break times, flexible start and end of shift, working without time pressure)
	Goodwill with regard to absence regulations and regular study days
Finances	Compensation for expenses/renumeration
	Additional financial support (e.g., free meals in the hospital, BAföG (state financial support for students with limited financial means), parents or partner for financial help, scholarship)
Personal matters	Motivation and interest (e.g., prospect of being employed in the PY clinic, retrieving and applying knowledge and skills, confirmation of or new insights into career choice or choice of specialist area)
	Positive work-life balance

Among other things, participants talked about a pleasant learning and working atmosphere that they appreciated, e.g., when they were able to make (topic) suggestions in the PY lessons or when they participated in what they wanted to learn or further improve. They also appreciated it when they perceived a gain in knowledge:

“One doctor kept doing little tests with me, nothing serious, it was actually just fun, but I took it very seriously and that was so helpful. We would do small examinations, so to say, practical and in written form, and I could really learn a lot from that.” (Interview 17).

Additionally, they liked doing interesting tasks, learning new techniques and being properly supervised with the opportunity to always ask questions. Several students considered it helpful to work independently and take responsibility in order to prepare for their future job:

“Sometimes, you are allowed to make your own decisions, and you just explain to the ward physician what you were doing and can ask them, if that’s okay. You’re really lucky to be able to do that.” (Interview 15).

Clear requirements and the communication of expectations beforehand as well as good equipment at work (e.g., their own workplace) were also perceived as resources. Finally, goodwill on the part of the supervisors with regard to the absence regulation and working times, regular study days and financial support seemed to help them a lot (e.g., state financial support, financial support from their parents).

Due to limited space a more detailed description of psychosocial resources and corresponding quotes from the students are shown in Appendix C.

### Proposed solutions

A selection of different solutions mentioned by the students sorted by main categories with a selection of suggestions and exemplary quotes can be found in Table 4.

**Table 4:** Proposed solutions (main categories with a selection of suggestions and exemplary quotes)

Categories	Suggestions	Exemplary Quotes
Learning and working environment	• More appreciation from superiors, nursing staff and patients	
Teaching	• Practical lessons instead of frontal teaching • Application-related topics of the PY teaching lessons • Stress management seminars • Interactive PY teaching • Structured training program (all PY students receive the same teaching and are no longer dependent on the commitment or goodwill of the supervising teaching physicians) • Preparation and skills seminars • Care of own patients (from admission to discharge) • Predefined learning objectives, e.g., in the form of voluntary logbooks or catalogs of learning objectives • Designated medical contact person in case of problems (one per ward) and a designated person responsible for teaching (control of the learning objectives to be achieved) • One-on-one teaching/mentoring • Better formation and training of supervising teaching staff (dealing with students, their integration into the ward team, general conditions of the PY such as working hours per week, tasks and learning objectives in the PY) • More initiative from teaching staff (more frequently taking on tasks)	“I think that the PY classes could be optimized. I would focus a bit on practical training and something like introduction to devices. So that you really get some training, no matter if it’s the software “Medico” or some medical devices like dialysis machines; there were no introductions and you just stayed away from everything, or you always had to ask others for help. And it would have been nice if you had had more guidance.” (Interview 6) “More like a mentor, exactly. So that when I’m on the ward and I have one or two patients I take care of, that there is another person talking care of them with me, someone who gets a bit involved and with whom you can exchange information. In other words, a designated contact person who also teaches you something, who guides you through things and shows you how everything works.” (Interview 10)
Work-related aspects	• Contact point for emotional stress • Take on medical activities only (no errands, patient transportation and nursing tasks) • More time to get to know work processes (accompaniment of a permanent supervisor during the initial period) • More responsibility and trust from superiors and therefore more opportunities to work independently • Stronger integration into everyday working life with firmly assigned tasks • Regular team meetings (so called team-time-out) • Introduction to equipment	“I would like to have certain groups where you can also bring in cases that affected you in a way; that is also often missing in the clinic for the doctors. And I think this would be actually cool, […] because it’s also the first time you come into contact with these issues.” (Interview 3)
Work organization	• Communication of the duties and rights of PY students (e.g., tasks and working hours) • Communication of supervisors’ expectations • Employment/internship contract with clearly defined working hours/week, rotation schedule and expense allowance • Appropriate distribution of PY students • Rotation schedules: rotations every 4–8 weeks (individual) • More flexibility in the allocation of PY tertials • Standardization of the PY • Own workstation with PC • Cross-clinic advocate for students in their PY	“Right now, I would like to have more access to, let’s say, all the different programs. I’d probably need to have three or four access points so that I could do some things more independently. I think it would be cool to consider PY students so that we don’t have to beg for access to a computer. Instead, we should just be able to access everything, and everything should be working. So that you could also take better care of your patients or think about them better. And simultaneously, you could be supervised and talk about it with the doctors.” (Interview 5)
Social interactions	• More team integration • More feedback in the PY • Make the role of practical year students better known to the public	“You should be integrated in the team as a PY student. And that you’re not addressed as “the PY student” but that you’re addressed by your real name.” (Interview 12)
Time	• Offer of voluntary night shifts • Clearly defined break times with colleagues (exchange) • More flexible working hours by relieving routine tasks, e.g., through a blood collection service • Fixed weekly working hours according to the study regulations • Absence regulation • More time for teaching, specialist training, preparation and follow-up of topics during the PY	“And I would like to have lunch breaks with others on a regular basis, so that you can also talk about things.” (Interview 20)
Finances	• Higher compensation for expenses	“I would improve the financial compensation so that people who depend on their salary can cover their living costs.” (Interview 20)

#### Teaching

PY students were in favor of the introduction of practical training (e.g., an introductory sonography course and a refresher course on the most common examination techniques in small groups) and theoretical seminars (e.g., concerning emergency situations). They would like to get to know diseases interactively with the appropriate diagnostic and therapeutic procedures using patient case examples. PY students would find seminars on topics such as dealing with stress, workloads, death and dying particularly useful as well as conflict situations with patients, supervisors and/or colleagues:

“There are so many content-related seminars, but I would like to see other things as well: how to handle stress, how to handle death or how do I cope with a high workload or with colleagues who are rude.” (Interview 2).

Students also suggested implementing one-on-one teaching or a mentor in the department who feels responsible for teaching.

#### Work-related aspects

PY students suggested a fixed place to go in case they experience emotional stress. They would like to be able to talk openly about stressful situations and receive professional support, e.g., from the hospital counseling (e.g., once a month). After tense, hectic or stressful situations (e.g., after unsuccessful reanimation), PY students would like a team-time-out of 5–10 min in which the situation could be discussed and analyzed retrospectively. Additionally, students would like to be trained in using technological devices, including PC software and medical devices such as sonography devices, ventilators and dialysis machines.

#### Work organization

PY students believed that the communication of supervisors’ expectations as part of an information event or a discussion with the supervisor responsible at the start of the rotation in the new department would be beneficial and could prevent miscommunication and enhance mutual understanding:

“I would have liked to have a conversation at the beginning of the PY with one of the senior physicians or someone else who is responsible for the students. So that this person tells you about their expectations, what they think your job is and what they want you to do. But also, that the PY student can talk about his or her wishes.” (Interview 1).

PY students also suggested an appropriate distribution of students (not more than two per ward) in order to minimize competition between students. In addition, they would like to have fixed rotation schedules and more individual opportunities to help shape the organization of PY tertials. Some students would like a second elective rotation, so that either the elective tertial is divided into two halves (2 months each) or tertials are replaced by quarters in one special field (internal medicine, surgery and two electives of 3 months each). The role and function of PY students should be made better known to the general population in order to facilitate contact with patients by increasing their trust in students. The nationwide standardization of the PY in terms of compensation, working hours per week and study days was also suggested by the participants:

“If I could decide, I would standardize the PY nationwide. Because I think it’s really annoying that, within the same city, you sometimes get paid, or you don’t get paid at all. Sometimes, you are supposed to work nine hours, sometimes six. […] It is completely random with regard to study days, payment and procedures. And I would try to standardize it so that it’s fair for everyone.” (Interview 2).

#### Social interactions

Students would like to be more integrated into the ward team (e.g., staff knowing the students’ names) and to be involved in the division of tasks. They also pleaded for more feedback during the PY (e.g., constructive feedback on a regular basis):

“I would like to change the feedback culture. I think there are some good approaches in other clinics who started piloting some projects. For example, that you can sit down with a doctor in the course of the week and you get profound feedback or that you always get a short feedback after certain situations or tasks.” (Interview 6).

#### Time

Students appreciated the opportunity to take part in night shifts in order to gain more knowledge and further improve their skills, which should be voluntary though. Students would want the number of 5–6 weekly working hours per day to be set in the study regulations, and hospitals should also adhere to that or allow for the division of hours: They suggested either working 5–6 h per day on five days of the week or keeping the regular working hours of resident physicians on four days of the week and then receiving one compensatory day per week (e.g., as a study day).

Regarding the absence regulations, students suggested separating sick days from vacation days. This would imply 30 vacation days with additional sick days (with a doctor’s certificate) if necessary:

“I think there should be vacation days and also sick days, just like for all other employees. So, that you need to bring a doctor’s certificate when you’re missing for more than three days in a row, for example. But you shouldn’t have any disadvantages because of that, and sick days shouldn’t be counted as days of absence.” (Interview 25).

In addition, the students would like to have 20 days of fixed study time between the end of the PY and the third state medical examination to better prepare for this final examination.

#### Finances

PY students also said that a higher allowance that corresponds to the minimum wage or is at least at the salary level of an apprentice would be appropriate and necessary in order to improve the conditions in the PY.

## Discussion

### Summary of the main findings

The present study was conducted to explore the experienced stressors, available resources and proposed improvements of PY students during the transition from mainly theoretical studies to clinical-practical training.

Mentioned stressors included a lack of skills and knowledge, poor supervision (i.e., lack of interest in learning success), routine tasks (i.e., lack of learning experiences due to repetitive work), lack of appreciation and a heavy workload. The resources included working with patients and their appreciation, a positive learning and working atmosphere, learning and knowledge acquisition, one-on-one supervision by a doctor in the ward, practical training as well as a positive work-life balance with sufficient leisure time. Some factors could be perceived as both a stressor and resource depending on their manifestation and/or each PY students’ preference. These factors were the quality of professional interaction with nursing staff and doctors, the degree of responsibility assigned, the level and number of assigned tasks and the working hours per week as well as the desired length of time on a ward (rotation plans). The PY students suggested better pay, a change in absence regulations as well as the possibility to take care of their own patients and structured PY teaching.

### Comparison with prior research

Our results are consistent with previous qualitative and quantitative studies among medical students (including PY students) [15, 33]. Stressors such as a persistent lack of time on the part of the supervising doctors (including lack of support, guidance, supervision and feedback [1, 16, 34, 35], and negative emotions (e.g., stress, anxiety, frustration and fear), evoked by taking on the role of a doctor [36], were described. Contact with pain, death and suffering as well as competitive thinking between medical students were also reported as challenging by medical students [4]. PY students also seem to experience some stressors that have been described for resident physicians, such as high work demands (workload, inefficient tasks, responsibility), concerns about patient care, poor work environment (relationships at work, supervision and support, lack of feedback) and poor work-life-balance [5] as well as lack of control [10].

Our findings related to resources are consistent with previous qualitative studies among medical students (including PY students). These include a supervision-oriented training curriculum with a defined contact person, a mentoring program, a distinctive feedback culture [1, 16] and integrated job-related intermediate examinations [16] as well as committed and supportive teaching doctors [1].

Stressors concerning, e.g., the inadequate compensation for the work performed, the strict absence regulations, the lack of a uniform structure in the training program and the fact that there is no fixed learning period between the end of the PY and the Third Medical State Examination were mentioned for the first time by participants in our study and seem to be exclusively related to the PY. Overall, we addressed both stressors and resources as well as potential solutions to improve conditions in the PY suggested by the students. Similar approaches have been used in other studies in Germany before, but they focused on medical students in general and dental students [1, 37].

The PY students in our study suggested a structured training program with interactive case-based learning in preparatory or skills seminars. Previous studies on interactive case-based learning showed that this motivating didactic learning tool contributes to the expansion of students’ knowledge and demonstrably improves performance results in the Objective Structured Clinical Examination (OSCE), which is a method of examination that allows for a standardized, reliable and objective evaluation [38]. Practicing clinical case studies with reflection and feedback has been found to be superior to a lecture-based approach [39]. A previous study in the form of an on-call simulation on a hospital ward reported that integrated learning and dealing with negative emotions can be useful in helping PY students feel better prepared to begin their careers as resident physicians [36]. A pharmacist-led prescribing program in which PY students made prescriptions, received interprofessional feedback from clinical pharmacists and took part in prescribing and calculation exercises improved the students’ confidence and prescribing skills [40, 41].

The students in our study also wished for more feedback during the PY (e.g., constructive feedback from the teacher responsible at least once a week). A prior study found that training with feedback from peers, standardized patients and a trainer resulted in a significant improvement in the medical students’ self-assessment of their communication skills [42]. Video-based supervision was not mentioned by participants in our study. Yet, in combination with feedback from supervisors, it can be an effective tool for improving clinical training in medical education [43]. This approach can help healthcare professionals develop and apply empathic and relational skills [44]. Video-based feedback sessions could be included in the final year training curriculum as they seem be associated with a perceived increase in self-perceived sympathy [45]. However, teaching doctors would need to make additional time for the regular feedback sessions with students.

Our participants would like to have a designated contact person for problems and a designated medical teaching doctor in terms of one-on-one teaching or mentoring. In order to be able to offer a one-on-one mentoring program, additional staff is of course required. As the establishment of such a mentoring program on each ward is time-consuming and costly for the teaching hospitals, its introduction seems challenging for the time being. However, research has confirmed that it is well received by PY students and allows teaching to be tailored to student expectations and interests [46]. A mentoring program with regular feedback may improve the practical medical skills and might better prepare PY students for their future work as resident physicians [18]. Students need support from teaching physicians in reflecting on their action, because teachers need to reflect on their own communication and interaction with patients and their own teaching behavior for students in order to be a good role model for empathic interaction with patients [47]. This enables the tutors to acquire comprehensive skills with regard to tutoring, but also in general with regard to the later role of the doctor [48].

### The framework conditions in German hospitals

The practical year is a special phase in the medical curriculum where students receive longer practical training in different hospitals and sections. Due to the clinical focus, certain issues might be related to the general framework conditions of the German healthcare system and the situation in German hospitals. It is possible that the learning atmosphere of students in hospitals is additionally influenced by (i) generational differences and potential conflicts, (ii) inadequate financing and skills shortage and (iii) a potential lack of interprofessional cooperation.

Firstly, three to four generations of physicians work together in clinics (Baby Boomers (*1946–1964), Gen. X (*1965–1980), Gen. Y (*1981–1995) and Gen. Z (*1996–2010)) [49]. The cooperation between the different generations and their partially differing view of work as well as the resulting demands on organizational structures and management styles thus becomes increasingly important. More experienced doctors (e.g., from the Baby Boomers) criticized less experienced doctors (e.g., from Generation Y) for their alleged lack of motivation, resilience, commitment and initiative and nurses and executives thought of Generation Y as not wanting to give themselves out and as not attaching such a great importance to work compared to Gen. X [49]. At the same time, doctors from Generation Y criticize a lack of understanding on the part of the more experienced for their idea of working hours and the balancing act between family and career [49, 50]. Our findings showed that PY students (Gen. Z) were motivated to work overtime if they experienced learning success (i.e., mean working hours/week = 37.1 instead of 20–25 h/week officially specified in the study regulations). This aligns with results of a previous study in which final-year students rated themselves highest in the competence area “Motivation” [51].

Secondly, German hospitals are under a lot of pressure due to a lack of professionals and rising costs [52]. This can potentially influence the capacity of doctors and supervisors and their general attitudes (e.g., too much stress for adequate training, no time to supervise others, too much workload to do teaching). Hospitals need proficient and skilled workers. Students in the PY can help reduce workload and take care of different tasks – provided they receive adequate supervision, guidance and training. Hospital staff could see this as a chance to receive support from colleagues in the making, so it might be useful to invest time in training them. Several participants in our interviews indicated that they use the PY to find their future employee or that they might stay in a hospital where the working conditions and atmosphere were pleasant. This should be taken in mind when guiding PY students, so that they are not seen as a burden but also as a relief and potential future colleagues on the ward.

Thirdly, there seem to be different levels of interprofessional cooperation and teamwork in German hospitals, even though it has been found that interprofessional teamwork is associated with a better perceived patient safety and that team interventions should be implemented in hospitals [53]. Improving interprofessional teamwork might also ameliorate the learning atmosphere and experience for PY students as this might also improve supervision.

### Demands of the Düsseldorf Medical Student Council

The inadequate working and training conditions during the PY are now increasingly portrayed in public media in Germany [54–56], and politicians have also become aware of the shortcomings in the practical training of medical students and now recognize the need for improvements. The four main demands of the Düsseldorf Medical Student Council and representatives of the Federal Representation of Medical Students in Germany [Bundesvertretung der Medizinstudierenden in Deutschland] (bvmd) correspond to the suggestions of participants in our study: a nationwide minimum compensation equal to the maximum BAföG rate (934€/month), separation of sick and vacation days, four weeks of study time before the Third Medical State Examination and a structured training curriculum with uniform teaching standards (including a mentoring program with regular feedback meetings, care for own patients under supervision, designated responsible physicians with experience, at least four hours of teaching per week and at least eight hours of self-study per week) [57], which further underlines the necessity for change.

### Strengths and limitations

One of the strengths of this study is the recruitment of participants from different age groups and genders to increase the likelihood that a wide range of possible opinions and experiences was captured. Additionally, we combined addressing stressors and resources and letting the students suggest potential solutions to improve study conditions in the PY. One limitation, however, is that the generalizability of our findings is limited as all participants were students at the HHU but worked in different hospitals at the time of data collection, which is a characteristic of qualitative research though. Correspondingly, we tried to establish transferability of data by describing our approach and decisions, by our sampling strategy and by adhering to the concept of data saturation. However, we did not adhere to other concepts of saturation (e.g., thematic or semantic), which might have further improved rigor. Some of the topics in our study may relate to specific conditions at teaching hospitals at our university (e.g., working conditions, compensation, teaching).

It is possible that only students who experience increased stress took part, because they were more interested in the research question. However, it could also be that students who are not overly stressed took part, because they might have had more time. Data collection by individual interviews is a strength of our study due to the increased anonymity in contrast to focus groups and thereby the better opportunities to raise unpleasant and sensitive topics (e.g., fears) or to express criticism.

SF had no prior knowledge of qualitative research methodology. The members of the research team gave her the best possible induction and prepared her for the data collection and analysis. Another limitation is the lack of pretests to thoroughly test the interview guide. Nevertheless, SF received feedback after the conduction of the first interviews. As part of her medical studies, SF already had some experience due to internships, so she may have prematurely assumed resources, stressors and suggested solutions and may have probed in less depth. However, the content analysis was carried out by two independent members of the research team (LG and SF), who worked intensively with the material. All four members of the research team, who have different backgrounds (AL: health scientist, TM: psychologist, LG: English Studies, and SF: medical student) reviewed the data several times and critically discussed the results. Notably though, SF has an interest in future improvements and the implementation of the proposed solutions, as she was about to be a PY student herself.

### Implications for further research and practice

The findings of our study can be used to improve the working and training conditions in the PY (student-centeredness with regard to the design of the PY) and to develop interventions. However, this could not only contribute to an improvement in clinical training, but also to staff recruitment and retention.

The results of this study should serve as a basis for further quantitative research to quantify and prioritize areas for intervention and experimental studies to test interventions that aim at improving the PY experience for students.

## Conclusion

Our qualitative study explored the stressors and the psychosocial resources experienced by PY students and suggested starting points for interventions. Several important aspects need to be implemented on a different level and require more actions from politics. Yet, our study shows that several starting points for improvements can also be achieved by every clinic and ward. It is therefore vital that every clinic and university is aware of the stressful conditions in the PY and tries their best to enhance study conditions, e.g., by involving students in decision-making, by active communication and by fostering a pleasant learning atmosphere.

## Supplementary Information

Supplementary Material 1.

Supplementary Material 2.

Supplementary Material 3.

## Data Availability

Data cannot be shared publicly, because the transcripts may contain sensitive information. The data may be obtained from the corresponding author upon reasonable request and provided that legal frameworks are not violated and that responsibilities and confidentially have been clarified.

## Abbreviations

BAföG: Federal Education and Training Assistance Act [Bundesausbildungsförderungsgesetz]
Bvmd: Federal Representation of Medical Students in Germany [Bundesvertretung der Medizinstudierenden in Deutschland]
COREQ: Consolidated criteria for reporting qualitative research
HHU: Heinrich Heine University
M3: Third medical state examination
PY: Practical year
OSCE: Objective structured clinical examination
