# Supplementary material for: The practical year: a qualitative study on stressors, resources and proposed improvements among medical students
Source: BMC Med Educ. 2025 Aug 23;25:1188. doi: 10.1186/s12909-025-07788-2 (PMC12374269; doi:10.1186/s12909-025-07788-2)
Supplement: Supplementary file 1 — Supplementary Material 1. [file 12909_2025_7788_MOESM1_ESM.docx]

**Appendix A: DÜSSELDORF CURRICULUM MEDICINE OF THE 3RD QUALIFICATION LEVEL**

The practical year (PY) corresponds to the 6^th^ year of the medical study at the Düsseldorf Medical School. Students are to complete the practical training in a clinical setting. Each year there is a spring cohort and a fall cohort and thus students start their practical year in mid-May or mid-November. Each PY cohort comprises approximately 100 students (1).

The practical year is split up in three 4-months training periods (i.e., “tertials”) which must be completed in different fields of medicine: one tertial must be completed in the surgical ward, including general, visceral and pediatric surgery, cardiovascular surgery as well as trauma and hand surgery (1). Another tertial must be completed in internal medicine. This includes endocrinology and diabetology, hematology, oncology and clinical immunology, intensive care medicine, cardiology, pneumology and angiology as well as rheumatology (1). The last tertial is the so-called elective tertial and it can be completed in any medical department. At the University Hospital Düsseldorf (UKD), Germany, electives include general medicine, anesthesiology, ophthalmology, dermatology, gynecology and obstetrics, ear, nose and throat medicine, pediatrics, medical microbiology, virology and infection epidemiology, oral and maxillofacial surgery, neurology, neurosurgery, nuclear medicine, orthopedics, palliative medicine, pathology, (clinical) pharmacology, psychiatry, psychosomatic medicine, radiology, (clinical) forensic medicine, radiotherapy and urology (1). Students can determine the order of the three tertials themselves (1).

All tertials can be completed in one or different hospitals in Germany (including the University Hospital Düsseldorf or academic teaching hospitals) or abroad (external institutions). It is possible to split tertials, which means that all tertials can generally be divided into two segments of eight weeks each. While PY students individually have to organize tertials abroad, PY training positions at university hospitals and teaching hospitals in Germany are mostly managed via an online portal (1).

1. Das Praktische Jahr. <https://www.medizinstudium.hhu.de/studienorganisation/studienjahre/praktisches-jahr-pj>. Accessed 01 May 2024.
