# Supplementary material for: The practical year: a qualitative study on stressors, resources and proposed improvements among medical students
Source: BMC Med Educ. 2025 Aug 23;25:1188. doi: 10.1186/s12909-025-07788-2 (PMC12374269; doi:10.1186/s12909-025-07788-2)
Supplement: Supplementary file 2 — Supplementary Material 2. [file 12909_2025_7788_MOESM2_ESM.docx]

**APpendix B: INTERVIEW GUIDE**

**“The practical year: a qualitative study on stressors, resources and proposed improvements among medical students“**

**A study by the Institute for Occupational, Social and Environmental Medicine at Heinrich Heine University Düsseldorf
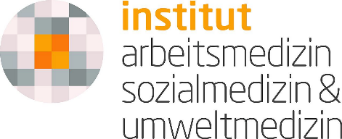
**

**Interview guide**

1. **Welcome**

Hello and welcome. First of all, I would like to introduce myself: My name is Syna Franck and I am a medical student in my 10th semester at Heinrich Heine University and a doctoral candidate at the Institute of Occupational, Social and Environmental Medicine at the University of Düsseldorf. Thank you very much for participating in this interview.

1. **Aim of the study**

In this study, I would like to investigate the burdens and negative aspects as well as the positive experiences in the PY. I am also interested in what you would improve or change.

At this point, it is important to say that there are no right or wrong answers. You are free to express your personal experiences, assessments and opinions.

The interview will take about 30 minutes.

1. **Confidentiality and anonymization of the data**

All data collected here will be treated in accordance with the current version of the General Data Protection Regulation. The conversation will be recorded on a tape recorder. I will NOT address you by name during the interview so that you remain anonymous. The tape recording will be transcribed afterwards by an external service provider and then destroyed immediately. Any information you provide about places or names (e.g., superiors) will NOT be recorded. The external service provider is subject to a confidentiality agreement.

You can end this interview at any time and without giving reasons. This will not result in any disadvantages for you.

1. **Declaration of consent**

I have received your declaration of consent. Have you received the document "Proband Receipt"?

1. **Questions?**

Do you have any questions before we get started?

I will turn on the tape recorder now and record a code at the beginning. This code will allow us to assign the interview later without your name.

1. **Switch on the dictation machine and record the code!**

To begin with, I would like to ask you a few questions about yourself.

| Gender | male female diverse |
| --- | --- |
| Year of birth (Age) |  |
| Semester |  |
| Specialty of the current rotation |  |
| Location of the current rotation |  |
| Working hours/ week, including overtime |  |

| **How often have you felt affected by the following complaints in the last 2 weeks? (PHQ2 + GAD-2)** | **Not at all** | **On single days** | **On more than half of the days** | **Almost every day** |
| --- | --- | --- | --- | --- |
| Little interest or pleasure in your activities | 1🞏 | 2🞏 | 3🞏 | 4🞏 |
| Dejection, melancholy or hopelessness | 1🞏 | 2🞏 | 3🞏 | 4🞏 |
| Nervousness, anxiety or tension | 1🞏 | 2🞏 | 3🞏 | 4🞏 |
| Not being able to stop or control worries | 1🞏 | 2🞏 | 3🞏 | 4🞏 |

**Interview questions**

| Introductory question about everyday working life | Please describe your current typical working day in the PY!  How have you experienced the PY so far? |
| --- | --- |
| Stressors | What stresses you in your day-to-day work?  What stressors have you experienced during your PY?  Have you experienced any emotional stress? |
| Resources | What positive experiences have you had?  What resources have you used during the PY? |
| Coping | What helps you personally to deal with the stress of the PY? |
| Proposed solutions | What would you like to change if you had the opportunity? What changes would you like to see? |
| Final questions | What expectations did you have of the PY? Were they fulfilled? What surprised you positively and negatively? How has your PY influenced your choice of specialist? |

1. **Conclusion of the interview**

From my point of view, we are done with the interview now. Finally, is there anything else you would like to say or add?

1. **Thank you for participating**

We are done with the interview now. I would like to thank you very much for talking to us! I will now stop the tape recording. Could you give me some final feedback? How did you experience the interview? What was positive and were any aspects missing?

1. **Final reflection**

Duration of the interview:

Atmosphere of the interview:

Conspicuous/special features:

What should be taken to the next interview/adapted?

**Note**: Each interview was introduced with an open introductory question and concluded with a final question. The purpose of these questions was to establish a trusting relationship with the participants and to provide an opportunity to address all topics relevant to them. However, these questions are not the focus of this paper and are thus not further elaborated.
