# Supplementary material for: The practical year: a qualitative study on stressors, resources and proposed improvements among medical students
Source: BMC Med Educ. 2025 Aug 23;25:1188. doi: 10.1186/s12909-025-07788-2 (PMC12374269; doi:10.1186/s12909-025-07788-2)
Supplement: Supplementary file 3 — Supplementary Material 3. [file 12909_2025_7788_MOESM3_ESM.docx]

**Appendix C: Psychosocial Resources**

Positive learning and working environment

Some PY students perceived the learning and working atmosphere as particularly positive when supervising assistant doctors and nursing staff showed interest in the students' learning success, were committed, integrated them well into the team, when the students’ work was appreciated by superiors and patients and when they had as much contact with patients as possible.

*“I’m always more motivated and happier when I’m able to work with patients a lot. For me, this is just so much fun, because they are usually very grateful.”*

Teaching

One-on-one teaching is a variant of teaching where each PY student is assigned to a permanent mentor, that is, a doctor who the students can accompany every day. This approach was mentioned as particularly positive by the students, since this mentor served as a permanent contact person for them. Students found the professional exchange instructive, as they were encouraged to think along and make suggestions regarding differential diagnoses, diagnostics and treatment options. They benefited from regular, short oral and written job-related intermediate examinations led by their mentor. The opportunity to also focus on what interested them and to help shape some of the teaching content was also perceived as a resource by PY students. This included adapting the rotation schedule within a tertial according to their own priorities and having flexible working hours (i.e., the choice between working in the outpatient clinic, on the ward or in the OR).

Work

Students found it useful to carry out clinical tasks such as taking blood, as they perceived a learning success after repeatedly performing them (e.g., taking blood samples from patients with difficult vein conditions without any problems). These routine tasks were only described as enriching if they are not the main activity during the working day (several hours). The PY students were particularly grateful when they were asked whether they would like to do routine tasks or whether they had enough time at that moment instead of simply being told to do them.

*“There were also two or three [blood samples] per day that the blood collection service didn’t manage, or the patients were not in their rooms at the time. And that was nice, because you had some routine, but you didn’t have to take blood from 20 patients. That really surprised me in a positive way.”*

*“I was allowed to examine and document the patients by myself and then I received feedback when I asked for it. I think that’s really good because you actually learn something when you make mistakes.”*

*“And that was really great in the outpatient department, it was extremely rewarding because I had my own patients there and I was able to talk about it before and after, either with an assistant doctor or sometimes even with the senior doctor, and we also discussed how to proceed and what other diagnostic issues I would consider.”*

The PY students expressed different opinions about their desired scope of responsibility. Some PY students were happy to have more responsibility for patients (e.g., registering examinations, initiating treatment, writing doctor's letters). Other PY students found it relieving to have little or no responsibility.

Organization

A clearly defined medical contact person or PY representative is perceived as an organizational resource for PY students. The hospital's PY representative takes care of all organizational issues (e.g., regarding rotation plans) and problems of the students, while the permanent medical teaching doctor/mentor of each department is responsible for the teaching and supervision of the individual student and works closely with them.

*“There are several levels of PY representatives. There is someone who takes care of organizational matters. She is from the secretariat, she is also PY representative. Then there is the medical one, i.e., the senior doctor, who is also involved and then there is an assistant doctor, who in fact comes to the ward from time to time. And this person also talks to us about how things are going, coordinates patients, discusses it with us and has a lot of contact with us overall. And it’s really motivating to know that someone is keeping an eye on you. And tells you: ‘If things aren’t going well, if someone doesn’t treat you well, please tell me.’ So that there is someone who says: ‘If anything goes wrong, I’m there for you and we’ll find a solution.”*

Social interaction

Students described intra- and interprofessional interaction between doctors and between doctors and nursing staff as positive if cooperation and communication with them seemed to be non-hierarchical and if the interaction was friendly and respectful. Students also appreciated when supervisors and colleagues valued their work.

*“Especially when it’s about vein accesses, blood sampling or bandage changes. We’ve done a lot that wasn’t actually our job, but we liked doing it because we want to learn it. And we were able to really support the nursing team and do a lot of their work, and they were so thankful, really appreciated it and were very kind and friendly.”*

Students were grateful when they received constructive feedback from their supervisors so that they could continue to improve in their work. Assistant doctors often integrated the students into the team and involved them in the contribution of daily tasks, which PY students viewed as positive.

*“I think especially assistants who have not been working for so long have a better connection to PY students, they also try to integrate them more, give them more tasks. I would say this is because they remember their time as PY students and know better what it was like. One of the assistants practically convinced me to start working there after all, because I was able to work like that. And I was really included and these were very positive experiences.”*

The relationships among PY students was perceived as a resource, especially during the initial period, because experienced PY students could introduce new ones to key tasks or programs (including software) or introduce them to new activities. The division of routine tasks was perceived to significantly reduce the workload for students. PY students understood that there was little room for questions in stressful situations or when there was a high workload. They also comprehended that nursing staff might show little interest in PY students, as they rotate to the next department after a few weeks. They also understood that they were expected to perform a high number of monotonous routine tasks when staff is missing. According to the PY students, supervisors knew that the students received low pay for their work, which is why they let them go home early sometimes.

Time

PY students did not perceive overtime as disruptive if they enjoyed their work, gained practical experiences or knowledge and received time off as a compensation. The number of working hours per week acceptable to students with a subjectively perceived intact work-life balance showed strong between-student variation. It was perceived as helpful, if PY students were able to make arrangements concerning their work time with supervisors or other PY students (e.g., leaving earlier in case of other appointments or swap shifts with other students). This was particularly helpful for students with children, as they may have had to take them to or pick them up from daycare or school. PY students found it particularly positive when supervisors offer them the opportunity to go home earlier when the work situation was quiet or allowed the students to decide for themselves when their shift ended. PY students felt relieved when their supervisors were indulgent regarding the formal absence regulations. Some of them discussed vacation and sick days within the department with their supervisors and did not have to take any official days off, which was appreciated and which also became evident in the stressor section.

*“Night shifts were also helpful because we had one-to-one teaching there. I really liked that and I would definitely keep mandatory night shifts for PY students. You have some really interesting cases there and you get the chance to examine patients yourself right away, and you also feel like being part of the team.”*

*“I have done a couple of shifts and I liked it because you were with a senior doctor and an assistant or with two assistants at times. They showed you much more, they also guided you way better. I was able to do about ten compression ultrasound examinations. And they were also supervising it and I could do it myself with each of the patients and that’s so useful. Because then, you can really do it and I thought that was really cool.”*

Finances

PY students were pleased to receive an allowance, even if it is small. Additional paid night shifts were viewed positively by PY students. In addition, students who had completed part of their final year in Switzerland reported that the pay there was significantly better than in German hospitals. PY students who received BAföG^[[1]](#footnote-1)^ felt grateful and relieved as they have no financial worries thanks to the state support.

Another resource for students was the financial support from their parents or partner.

*“Luckily, my parents support me financially, and they still do that even though I receive compensation. So this means I don’t have any financial trouble even though I don’t earn much money.”*

Some PY students received monthly financial support through a scholarship (e.g. [Deutschlandstipendium], foreign scholarship). This enabled the PY students to complete part of their PY abroad without having to worry about financial matters. PY students who live in a shared flat or with their partner found this a financial relief.

Personal matters

PY students felt most motivated in their elective tertial, as they were usually most interested in their clinical area of choice and may complete this part of their PY in the hospital where they wanted to work as a junior doctor after their final examination. Students took the opportunity to make important contacts in this time, e.g., for their application (especially superiors) and tried to make a good impression on staff (e.g., through extensive preparation of ward rounds and/or overtime). On the other hand, hospitals also seemed to recognize the opportunity to present themselves as an attractive employer and thus recruit new employees. Some students reported that they were so impressed by the positive working environment in specific departments, among other things, that they wanted to apply there after graduation.

*“It’s an individual issue, but I think it’s calming to know that becoming a doctor is the right thing for me. You just have to choose the right medical specialty and it’s good that there are also very nice superiors who don’t yell at you for making a mistake. So, that you’re really allowed to make mistakes.”*

*“I think you really want it to start now. But this is actually what the PY is about. You should feel adequately prepared so that you want to start working and also know how to do the job.”*

1. state financial support for students with limited financial means [↑](#footnote-ref-1)
